# In Vitro Polyploid Induction of Highbush Blueberry through De Novo Shoot Organogenesis

**DOI:** 10.3390/plants11182349

**Published:** 2022-09-08

**Authors:** Federico Marangelli, Vera Pavese, Giuseppe Vaia, Michela Lupo, Muhammad Ajmal Bashir, Valerio Cristofori, Cristian Silvestri

**Affiliations:** 1 Department of Crop Transformation, NIAB, 93 Lawrence Weaver Road, Cambridge CB3 0LE, UK; 2 Dipartimento di Scienze Agrarie, Forestali e Alimentari (DISAFA), Università degli Studi di Torino, Largo Paolo Braccini 2, Grugliasco, 10095 Torino, Italy; 3 Dipartimento di Scienze Agrarie e Forestali (DAFNE), Università della Tuscia, Via San Camillo De Lellis s.n.c., 01100 Viterbo, Italy

**Keywords:** *Vaccinium corymbosum* L., adventitious shoot regeneration, colchicine, oryzalin, polyploidy

## Abstract

Polyploid induction is of utmost importance in horticultural plants for the development of new varieties with desirable morphological and physiological traits. Polyploidy may occur naturally due to the formation of unreduced gametes or can be artificially induced by doubling the number of chromosomes in somatic cells. In this experiment, a protocol for in vitro polyploid induction of highbush blueberry (*Vaccinium corymbosum* L.) leaf tissues was studied by using different concentrations of colchicine and oryzalin. Oryzalin was found to be highly toxic to this species, while the adventitious shoot organogenesis media enriched with 25 and 250 µM colchicine was able to induce polyploidization, with significant differences among the treatments used. Higher concentrations of both antimitotic agents led to the browning and death of the leaf tissues. The polyploids obtained showed several morphological differences when compared with the diploid shoots. Flow cytometry analysis was used to confirm the ploidy level of the regenerated shoots, demonstrating that a total of 15 tetraploids and 34 mixoploids were obtained. The stomatal sizes (length and width) of the tetraploids were larger than those of the diploids, but a reduced stomatal density was observed as compared to the controls. These shoots will be acclimatized and grown until they reach the reproductive phase in order to test their potential appeal as new varieties or their use for breeding and genetic improvement.

## 1. Introduction

The highbush blueberry (*V. corymbosum* L.) is a species with escalating production worldwide and a commercially valuable minor fruit crop in Italy since it is a much sought-after species used to differentiate crops, utilize marginal lands and meet the increasing consumer demand. The potential uses of blueberries are manifold: from fresh consumption to processing for juices, jams, and even the pharmaceutical and cosmetics industries, since it is one of the richest sources of antioxidants with high potential to reduce the incidence of several degenerative diseases. Breeding of the highbush blueberry is focused mainly on low-chilling requirements, superior fruit quality [1], increased shelf-life, and tolerance/resistance to the most important pests and diseases.

After a relatively recent domestication, conventional breeding programs of the highbush blueberry continue to exploit its wide phenotypic diversity in order to meet the increasing market demands. Conventional breeding by germplasm selection or intra- and interspecific hybridization has been widely used for the development of new varieties of highbush blueberry [1,2,3,4,5]. However, the efficiency of traditional breeding can be improved by using marker-assisted selection (MAS), but it remains a challenge for crops such as the highbush blueberry, characterized by polyploidy and a highly heterozygous genome [6]. The genetic improvement of this species has also been performed through genetic engineering [7,8,9,10,11,12], and more recently, some protocols on the use of genome editing have been published [2,13]. Despite this progress, no transgenic varieties have been released for commercialization yet [2] because of non-science-based concerns and laws regulating GMOs [14].

Therefore, tissue culture-mediated breeding techniques have become part of the commonly used methodologies for obtaining new varieties (of agricultural interest), which have the advantage of accelerating and enhancing classical breeding programs. These techniques are mainly based on somaclonal variations obtained through appropriate protocols from cells and tissues grown in vitro. In blueberries, various PGRs such as zeatin, zeatin riboside, zeatin trans-isomers, 2iP, TDZ and NAA are routinely used for inducing the regeneration of *Vaccinium* species. In the literature, the results obtained confirm that zeatin and zeatin riboside resulted superior to other cytokinins in the induction of direct shoot organogenesis in highbush blueberry [15,16]. Therefore, having an efficient regeneration protocol (i.e., de novo shoot organogenesis or somatic embryogenesis) makes it possible to artificially exploit this phenomenon, either by subjecting cells to biotic and abiotic stresses that cause the cell to ‘mutate’ for their survival or by selecting cells that can tolerate stress. In addition, regeneration events can also be exploited by subjecting explants to chemical agents that produce mutations and/or disturbances in cell division, such as mutagenic agents, and are able to induce polyploidy [17]. Induced mutagenesis often creates phenotypic and genotypic chimeric mutants, also called “sports” [18]. Adventitious organogenesis could represent a strategy to reduce/eliminate the chimerism, since the chimerism cannot be excluded by sexual propagation and segregation [19].

In this context, there is growing interest in obtaining plants with a higher level of ploidy, which could lead to phenotypic changes in plants as a result of alterations in gene expression [17]. Moreover, polyploidy has a significant impact on the morphology and physiology of newly formed offspring. Compared with the corresponding diploids, polyploids tend to have larger cells, which results in the enlargement of single organs, such as leaves, flowers and seeds. Physiological traits such as plant height, growth rate, flowering time and fertility can also be altered by polyploidization [20]. Recent studies have shown that the large phenotypic differences between polyploids may be related to subtle changes in gene expression rather than a substantial change in transcription [21,22,23]. In addition, tetraploid plants very often show phenomena of ‘gigantism’ that are highly desirable, particularly when they occur at the level of the fruits. In other species, the development of polypoid genotypes has also been pursued with the aim of employing them as rootstocks suitable for reducing plant size [24] or for increasing resistance to water stress by increasing the constitutive production of abscisic acid [25], or as cultivars, which are often characterized by a reduced growth habit and are ultimately more adaptable to new plantings that prefer high-density plantation.

In this study, we evaluated the efficiencies of antimitotic agents in inducing polyploidy in highbush blueberry, obtaining polyploid and mixoploid shoots. This experiment aimed to develop a protocol for in vitro polyploid induction and to identify diverse breeding materials of highbush blueberry with improved traits that can be used as parent material in its future breeding programs.

## 2. Results and Discussions

In this study, the successful induction of artificial polyploidy in *V. corymbosum* L. by using colchicine and oryzalin was achieved. Shoots of the M5 variety were successfully established in vitro and, after three subcultures, they were used as explants for the experiments.

**Figure 1:**
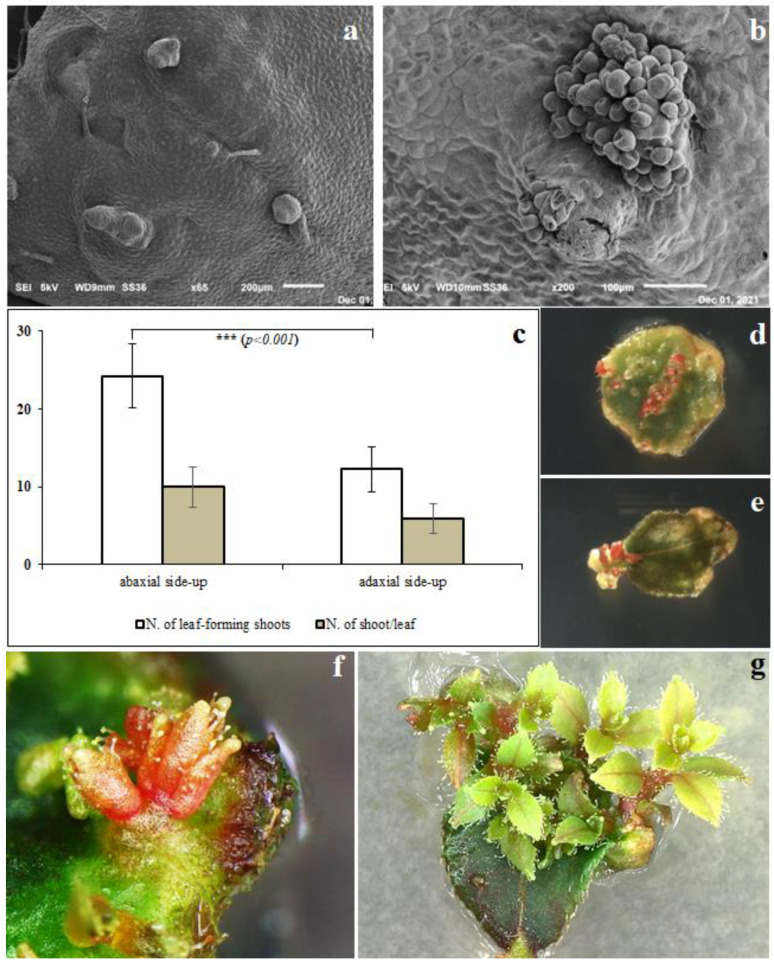
(**a**) Leaf tissues observed under a scanning electron microscope (SEM) after 5 days in culture medium with evidence of single or (**b**) grouped bud primordia. (**c**) Effect of explant orientations (abaxial or adaxial side up) on in vitro shoot organogenesis in “M5” highbush blueberry. De novo shoot organogenesis on the leaf surface closer to the vein (**d**) or to the petiole (**e**). First organogenesis from mutagen-free medium (**f**) and developed adventitious shoots (**g**).

The incidence of de novo shoot organogenesis was observed just 5–6 days after the explants were placed in the culture medium, as validated with SEM images, in which single (Figure 1a) or grouped (Figure 1b) bud primordia were observed, confirming that the induction and differentiation phases in this species take place very quickly. Furthermore, the highest shoot induction efficiency was observed in the leaf cultured with the abaxial side up, as compared to the leaf cultured with the adaxial side up. This criterion resulted in lower adventitious shoot induction in terms of the number of leaves forming shoots and not in terms of the number of shoots per leaf (Figure 1c). Different responses in terms of shoot regeneration, depending on the orientation of the explants, have been reported in the ‘Red Pearl’ and ‘Ozarkblue’ varieties of highbush blueberry [26], cranberry (*Vaccinium vitis idaea* L.) [27,28] and rabbiteye blueberry (*V. virgatum*) [29]. Surprisingly, in contrast to most of the other species, shoot organogenesis did not occur frequently from the wounds but on the whole leaf surface, particularly closer to the veins (Figure 1d) and leaf petiole (Figure 1e). These results were also observed by [30], who described two different patterns during the de novo organogenesis processes of various cultivars of southern highbush blueberry: pattern I, where regenerants emerged mainly from the portion closer to the cutting area, and pattern II, where regeneration occurred directly from the entire leaf blade. In general, they did not observe the callus-mediated de novo organogenesis using the regeneration medium described here.

Based on these preliminary results, the leaves used for polyploidy induction were cultured with the abaxial side up and were used to evaluate the effect of different concentrations of both antimitotic mutagens for the induction of polyploidy studies (Table 1).

As shown in Table 1, the number of leaves showing organogenesis was ˃80% in the mutagen-free media (control), with an average number of regenerated buds per leaf of 8.2. In colchicine-enriched media, at both concentrations of 25 and 250 µM, the number of leaves showing regeneration and the number of buds per leaf were significantly lower with respect to the control: in particular, only 36.5% and 4.5% of the leaves, respectively, showed shoot organogenesis and, similarly, the number of regenerated buds per leaf was inhibited by the presence of colchicine, with average values of 4.2 and 1.3 buds per leaf, respectively. The colchicine concentration of 500 µM was too high, completely inhibiting shoot organogenesis (Table 1) and causing tissue necrosis within 36 h of the start of the experiment (data not shown).

Leaves cultured on colchicine- and oryzalin-free media started showing the first organogenesis events just after 9–10 days in culture medium (Figure 1f), whereas the explants cultured in media enriched with colchicine showed the first visible buds about 4–5 days later than the controls. After 30 days from the start of the experiment, when the newly formed shoots had developed 2–3 nodes (Figure 1g), the shoot regeneration rates and the number of shoots per explants were determined. Interestingly, while the leaf tissue treated with different concentrations of colchicine showed shoot organogenesis, albeit with a significantly lower regeneration rate than the control, the oryzalin-treated leaves did not show any regeneration, regardless of the concentration.

Furthermore, colchicine significantly inhibited the shoot organogenesis of the explants, in line with what has been observed by other authors [17,31,32,33]. Finding a suitable concentration of antimitotic agent is crucial for the success of the experiments, since it depends not only on the species but also on the genotype. Surprisingly, no results were obtained with the use of oryzalin, even at low concentrations, unlike for other species where the use of oryzalin was also found to be less phytotoxic than colchicine treatments. In [34], the authors reported that for passion fruit (*Passiflora edulis*), the highest concentration used (30 μM) applied for 15 days resulted in 98% viable explants, with a tetraploid regeneration frequency of 2%. However, contrary to our results, colchicine has been reported to be more phytotoxic than oryzalin in several species [35,36,37].

**Figure 2:**
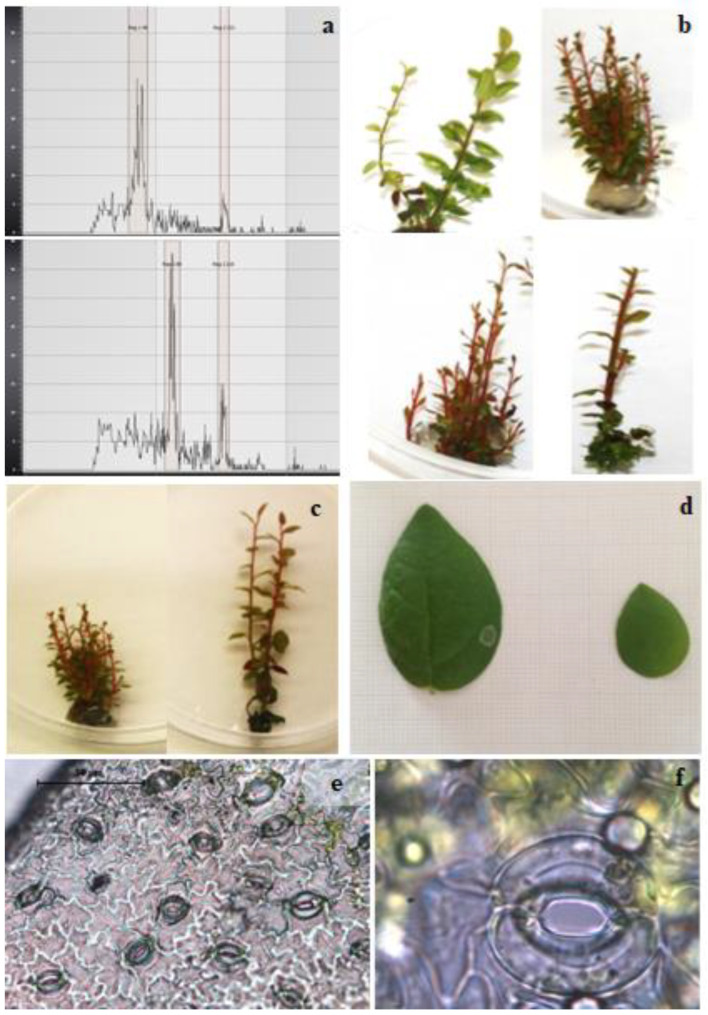
(**a**) Histograms of the flow cytometry of diploid (top) and tetraploid (bottom) shoots. (**b**) Morphological differences among mixoploid shoots. (**c**) Differences between diploid (left) and tetraploid shoots. (**d**) Fully expanded leaf blade of a tetraploid (left) and diploid (right) shoot. (**e**) Stomatal density of the abaxial surface of leaf derived from diploid shoots. (**f**) Stomata from a tetraploid observed under an optic microscope (100×).

At completion of the experiments, a total of 409 regenerated shoots had been obtained (165 from both 25 and 250 µM colchicine treatments) (Table 1). The ploidy level was also ascertained, and a total of 15 tetraploids and 34 mixoploids (of these, 31 were from the treatment at 25 µM and 3 were from the 250 µM colchicine treatment) were recorded. For the confirmation of ploidy level, Figure 2a shows the histograms of a diploid (2*n* = 48) shoot and a representative tetraploid (2*n* = 96) shoot. The tetraploid induction rates are presented in Table 1: both (25 and 250 µM) colchicine treatments produced tetraploid shoots, and the ploidy induction rate ranged between 7.8 and 11.0, which was significantly higher in the medium enriched with 250 µM colchicine as compared to the 25 µM treatment. Interestingly, in the treatments with 25 µM colchicine, about 30% of the regenerated shoots were mixoploids, in contrast to those derived from 250 µM colchicine, where only about 5% of the shoots showed mixoploidy. Both polyploids and mixoploids showed some differences in phenotypic traits, and some representative plants are shown in Figure 2. In particular, Figure 2b shows the different morphological traits of some representative mixoploid shoots, while Figure 2c shows the morphological differences between diploid and tetraploid shoots. Mixoploid shoots showed different morphology among them and within shoots of the same genotype due to the presence of tissues with different cytotypes (Figure 2b), while the diploid and tetraploid shoots (Figure 2c) appear uniform, with differences in terms of growth: tetraploid shoots were taller than the diploid ones, although the tetraploids developed less shoots per explant compared to the diploids. The results obtained in the present work showed that the best concentration for polyploid induction of highbush blueberry is 250 µM, since it allowed for some tetraploids but with a low incidence of mixoploid shoots. Higher concentrations of colchicine resulted deleterious to polyploid induction and shoot regeneration, as reported also in others’ experiments with different species and explant types used [17,37,38,39]. According to the literature, mixoploids are often obtained during the artificial induction of polyploids in vitro, and they are usually regarded as a failure or undesirable by-products of polyploidization studies due to the occurrence of a mutation/change at the chromosome level in cell layer I or II of meristematic tissues, and they do not yield stable polyploids [40]. For this reason, dose applied and time of exposure are crucial to avoid the appearance of mixoploid shoots [17,41]. In this regard, efforts have been made to eliminate them through deliberate means, namely mechanical isolation of putative polyploids [42,43], shoot regeneration using nodal segments [44] and repeated subcultures of apical buds [45]. Moreover, the problem of mixoploidy could also be overcome through a second round of adventitious shoot organogenesis that could enhance the occurrence of stable polyploids, as reported by [2] in experiments of genome editing in highbush blueberry.

To better understand the stomatal traits with respect to the ploidy level, stomatal length, width and density were compared, and the results are listed in Table 2. Moreover, the leaf blades of the tetraploid were larger than those of the diploid (Figure 2d). The stomatal density observed was very high in the leaf of diploids (854.6 stomata/mm^2^) (Figure 2e). On the other hand, the average length and width of the stomata in tetraploid shoots were higher than the diploid ones (control), but an increase in stomatal size (Figure 2f) corresponds to a decrease in density, since the tetraploid shoots showed a density of 491.2 stomata/mm^2^. These findings are consistent with the results observed in most of the other species after polyploidization, such as bigger leaves and different phenotypes. In any case, the vegetative and reproductive traits need to be analyzed under field conditions. The effects of polyploidization could result in the enlargement of the fruits, long-lasting flowers, differences in terms of pest and disease resistance and less barriers for hybridization [46,47], and the breeding and genetic improvement can provide several advantages to improve the agronomic traits of important species. As previously reported, autotetraploidization has profound effects on morphological and physiological events and modifies both primary and secondary metabolism in Ponkan mandarin fruit. Moreover, it reduced the transcription of citric acid transport-related and utilization-related genes in fruit [48]. In addition, Tan et al., 2017 [49], observed that polyploidization had a significant but relatively limited influence on the accumulation of metabolites in three citrus species, i.e., red tangerine (*Citrus reticulata* Blanco), trifoliate orange (*Poncirus trifoliata* L. Raf.) and precocious trifoliate orange (*P. trifoliata*). They conclude that primary metabolism takes priority over secondary metabolism in doubled-diploid plants to relieve the “genomic stress” encountered during the early stages of genome doubling, probably to promote vitality and growth of plants.

## 3. Materials and Methods

### 3.1. Plant Material

The plant material used in our study was a clonal selection named M5. Uniform twigs were collected from the greenhouse and rinsed under running tap water to remove the dirt after leaf removal. After washing, the twigs were immersed in aqueous solution containing 250 mg/L of ascorbic acid, 250 mg/L of citric acid and 0.1% PPM ^®^ for 40 min [50]. Then, the shoot apices and the nodal segments with axillary buds were sterilized with an aqueous solution of commercial bleach (20%) containing a few drops of tween 20 ^®^ for 20 min and rinsed three times with sterile distilled water under a laminar flow hood. The explants were cultured for 15 days in a hormone-free WPM medium [51], at pH 5.4.

Sterilized shoots were transferred to the proliferation medium, containing WPM medium including vitamins, 3% sucrose, 250 mg/L of L-glutamine, 2.28 µM zeatin riboside (ZR) and 0.25 µM indole-3-butyric acid (IBA).

### 3.2. Adventitious Shoot Organogenesis and Polyploid Induction I

To induce polyploidy, fully expanded leaves were collected from 21-day-old in vitro growing shoots and cultured in the adventitious shoot organogenesis medium (SOM) consisting of WPM medium including vitamins [51], 3% sucrose and 11.4 µM zeatin riboside (ZR).

A stock solution of colchicine (10,000 mg/L) was prepared by dissolving 100 mg of colchicine powder in 10 mL of sterile deionized water and filter-sterilized with a 0.22 μm Millipore filter, and then added to the above-described SOM medium to reach the final concentrations of 25, 250 and 500 μM. Similarly, a stock solution of oryzalin (2500 mg/L) was prepared by dissolving 25 mg of powdered oryzalin in 1.5 mL of dimethyl sulfoxide (DMSO), and brought to a final volume of 10 mL with sterile deionized water and then filter-sterilized following the same procedure mentioned above. The stock solution of oryzalin was also added to the SOM medium to obtain the final concentrations of 25 and 250 μM. Twenty-five milliliters of the media containing different concentrations of colchicine and oryzalin were poured into Petri dishes (Ø 96 mm), and 26 leaves were cultured in each plate. Six plates were used for each treatment. Before the setup of the experiment, a test was carried out with the aim of investigating the organogenetic ability of the explants with adaxial or abaxial side up. The Petri dishes were kept in the dark for 10 days, and then, the plates were transferred to oryzalin- and colchicine-free media and then exposed to the light condition, with a 16 h photoperiod with a PPFD of 40 μmol m^−2^s^−1^ at 24 ± 1 °C.

During all the experiments, the plates were regularly checked for contamination and under the stereomicroscope for the appearance of the first buds.

At the end of the experiments, the percentage of explants forming shoots and the number of adventitious shoots were determined. Regenerated shoots showing atypical visual morphology (leaf thickness, shape and size) were selected as possible polyploids or mixoploids.

### 3.3. Media Preparation and Sterilization Conditions

The pH of the media was adjusted to 5.4 with 1N HCl or 1N KOH before autoclaving at 121 ± 1 °C, 1 atm for 20 min, and all the media were supplemented with 0.6% Plant Agar^®^. All chemicals were purchased from Duchefa Biochemie NL (Haarlem, the Netherlands).

### 3.4. Ploidy Evaluation

Flow cytometric analysis of putative polyploid shoots was performed according to [52], after the removal of leaf trichomes as described by [53] by using Ploidy Analyzer PA I (Sysmex Partec, Germany). The leaflets were chopped with a sharp razor blade while submerged in the nuclei extraction buffer from CyStain™PI Absolute P (Sysmex Partec GmbH, Görlitz, Germany). The homogenate was filtered through a 30 μm nylon mesh to remove debris and then stained with RNAse A and propidium iodide (PI) according to the manufacturers’ instructions. To assess ploidy levels of the putative polyploids, *Allium cepa* was used as an internal standard.

### 3.5. Electron Microscopy and Stomata Traits

For scanning electron microscopy (SEM), leaves used for adventitious shoot organogenesis were collected and cut in transversal sections, and then treated by following the protocol described by [54]. The observation was made by a JEOL JSM 6010LA scanning electron microscope. For the analysis of stomata traits, fully expanded leaves from month-old growing shoots were collected. For the stomatal size analysis, 30 stomata from each leaf were randomly measured. Leaves were wounded and immersed in water overnight, and the epidermal layer was peeled off and placed on a glass slide with water droplets, covered with a cover slip and observed under a light microscope (DM4000 B, Leica, Germany) at a magnification of 40×.

### 3.6. Statistical Analysis

The data were subjected to analysis of variance (ANOVA) performed using the SPSS software, version 20.0. Percentage data were transformed as the arcsine of the square root of p/100 before analysis of variance. Fisher’s test and Student’s test were used for mean separation and to provide homogeneous groups for the means (at *p* ≤ 0.01).

## 4. Conclusions

A suitable protocol for shoot regeneration from the leaf tissue of highbush blueberry has been obtained using the WPM medium including vitamins [51], 3% sucrose and 11.4 µM zeatin riboside (ZR). We conclude that the most effective method for obtaining tetraploids consists in using 250 µM colchicine for ten days and then transferring the explants to the same colchicine-free medium, allowing for a tetraploid induction rate of about 11% and reducing the induction rate of mixoploids. The results obtained confirm that the plantlets with different ploidy levels show modified morphological traits, especially leaf traits. It would be interesting to set up new experiments to better understand what range of concentrations of oryzalin is able to induce mutagenesis/polyploidy in highbush blueberry.

As far as we know, this is the first evidence of novel and successful induction of polyploidy in highbush blueberry. The genetic material developed in the present work is being used for further selection and crosses.

## Figures and Tables

**Table 1:** Effect of colchicine concentration on somatic tissue chromosome doubling in *Vaccinium corymbosum* L. Data are presented as mean ± S.D. The means denoted by different letters are significantly different (Fisher’s test, *p* < 0.01). Data on oryzalin treatments have not been reported in the table as no regeneration has been achieved.

Treatment Colchicine (µM)	Leaf-Forming Buds (%)	No. of Buds per Leaf	No. of Regenerated Shoots	No. of Tetraploids ^x^	No. of Mixoploids ^y^	Tetraploid Induction Rate (%) ^z^
0 (Control)	81.5 ± 6.1 a	8.2 ± 1.6 a	244	0	0	-
25	36.5 ± 7.8 b	4.2 ± 0.7 b	102	8	31	7.8 ± 1.2 b
250	4.5 ± 3.2 c	1.3 ± 0.4 c	63	7	3	11.0 ± 1.4 a
500	-	-	-	-	-	-

^x,y^ Data represent the sum of three replicates. ^z^ Data represent the mean ± SD of three replicates.

**Table 2:** Differences in stomatal traits of diploids and tetraploids of *Vaccinium corymbosum* L. var. M5. Data are presented as mean ± S.D. The means denoted by different letters are significantly different (Student’s test, *p* < 0.01).

Ploidy Level	Stomata Length (µm)	Stomata Length (µm)	Stomata Density (N/mm^2^)
Diploid	11.91 ± 0.63 a	5.2 ± 0.56 a	854.6 ± 22.1 a
Tetraploid	18.59 ± 0.51 b	12.45 ± 1.12 b	491.2 ± 52.1 b

## Data Availability

Not applicable.
